# Preoperative environment enrichment preserved neuroligin 1 expression possibly via epigenetic regulation to reduce postoperative cognitive dysfunction in mice

**DOI:** 10.1111/cns.13777

**Published:** 2021-12-09

**Authors:** Jia Min, Zhongmeng Lai, Hui Wang, Zhiyi Zuo

**Affiliations:** ^1^ Department of Anesthesiology University of Virginia Charlottesville Virginia USA; ^2^ Department of Anesthesiology First Affiliated Hospital Nanchang University Nanchang China; ^3^ Department of Anesthesiology Fujian Medical University Union Hospital Fuzhou China; ^4^ Department of Anesthesiology General Hospital of Ningxia Medical University Yinchuan China

**Keywords:** environment enrichment, histone deacetylase, neuroligin 1, postoperative cognitive dysfunction

## Abstract

**Aims:**

Postoperative cognitive dysfunction (POCD) is a common and significant syndrome. Our previous studies have shown that surgery reduces dendritic arborization and spine density and that environment enrichment (EE) reduces POCD. Neuroligin 1 is a postsynaptic protein involved in the formation of postsynaptic protein complex. This study was designed to determine the role of neuroligin 1 in the protection of EE against POCD and the mechanisms for EE to affect neuroligin 1 expression.

**Methods:**

Eight‐week‐old C57BL/6J male mice with or without EE for 3, 7, or 14 days had right carotid artery exposure under isoflurane anesthesia. An anti‐neuroligin 1 antibody at 1.5 µg/mouse was injected intracerebroventricularly at one and two weeks before the surgery. Mice were subjected to the Barnes maze and fear conditioning tests from one week after the surgery. Cerebral cortex and hippocampus were harvested after surgery.

**Results:**

Mice with surgery had poorer performance in the Barnes maze and fear conditioning tests than control mice. EE for 2 weeks, but not EE for 3 or 7 days, improved the performance of surgery mice in these tests. Surgery reduced neuroligin 1 in the hippocampus. Preoperative EE for 2 weeks attenuated this reduction. The anti‐neuroligin 1 antibody worsened the performance of mice with surgery plus EE in the Barnes maze and fear conditioning tests. Surgery increased histone deacetylase activity and decreased the acetylated histone in the hippocampus. EE attenuated these surgery effects.

**Conclusion:**

Our results suggest that preoperative EE for 2 weeks reduces POCD. This effect may be mediated by preserving neuroligin 1 expression via attenuating surgery‐induced epigenetic dysregulation in the brain.

## Graphical abstract

Postoperative cognitive dysfunction (POCD), a common condition after surgery, may be mediated by reduced neuroligin 1 possibly via histone deacetylase activation. Environment enrichment blocked these biochemical effects to reduce POCD in mice.

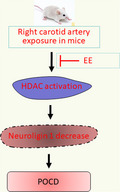

## 1 INTRODUCTION

Postoperative cognitive dysfunction (POCD) affects millions of patients each year in the USA and is associated with poor clinical outcome.^1^, ^2^, ^3^ Many animal models have been established for POCD research,^4^, ^5^, ^6^, ^7^ and neuroinflammation appears to be a fundamental neuropathological process for POCD.^4^, ^8^, ^9^, ^10^ However, general inhibition of inflammation may not be a good approach for reducing POCD during the perioperative period due to the concern of potential side effects of the therapy, such as wound healing impairment and infection. Up till now, effective and practical interventions for POCD have not been established.

Environment enrichment (EE) can improve cognitive functions.^11^ To search for non‐pharmacological methods, we and others have shown that EE reduces POCD in rodents.^12^, ^13^, ^14^ Our previous studies are focused on applying EE after surgery^12^, ^14^ because of the thinking that patients after surgery in the hospital may be more compliant to practice EE with the help of healthcare providers than patients at home before the surgery. One study has shown that EE for 2 weeks before surgery attenuates POCD in rats.^13^ However, the length of preoperative EE for it to be effective and the mechanism for this effect have not been determined.

Neuroligin 1 is a cell adhesion protein that is expressed in the postsynaptic membrane of excitatory synapses.^15^, ^16^ Neuroligins interact with presynaptic proteins to form synapses and recruit and stabilize key synaptic components into the postsynaptic membrane.^15^, ^16^ Neuroligin 1 is critical for long‐term potentiation development and is critical for the activity‐dependent maturation of excitatory synapses.^17^, ^18^ Mice with neuroligin 1 knockout have impaired spatial memory and increased repetitive behavior.^19^ These mice have a significant impairment in sleep‐wake cycle regulation.^20^ Patients with Alzheimer's disease or mice with Alzheimer's disease have decreased neuroligin 1 in their brain.^21^ However, the role of neuroligin 1 in POCD is not clearly defined.

Our previous study has shown that surgery increases histone deacetylase (HDAC) activity and decreases growth factor expression in the brain.^7^ Increased HDAC can lead to decreased neuroligin 1.^22^ Thus, we hypothesize that surgery reduces neuroligin 1 expression, which contributes to the development of POCD, and that EE attenuates this reduction of expression via blocking epigenetic dysregulation after surgery to improve learning and memory. To test these hypotheses, mice were subjected to carotid artery exposure, a surgical procedure that does not affect the motor function. Normal motor function is needed for performing learning and memory tasks.

## 2 MATERIALS AND METHODS

All experimental protocols were approved by the Institutional Animal Care and Use Committee of the University of Virginia (Charlottesville, VA). All animal and experimental procedures were carried out in accordance with the National Institutes of Health Guide for the Care and Use of Laboratory Animals (NIH publications number 80–23) revised in 1996.

### 2.1 Animal groups and housing

**FIGURE 1:**
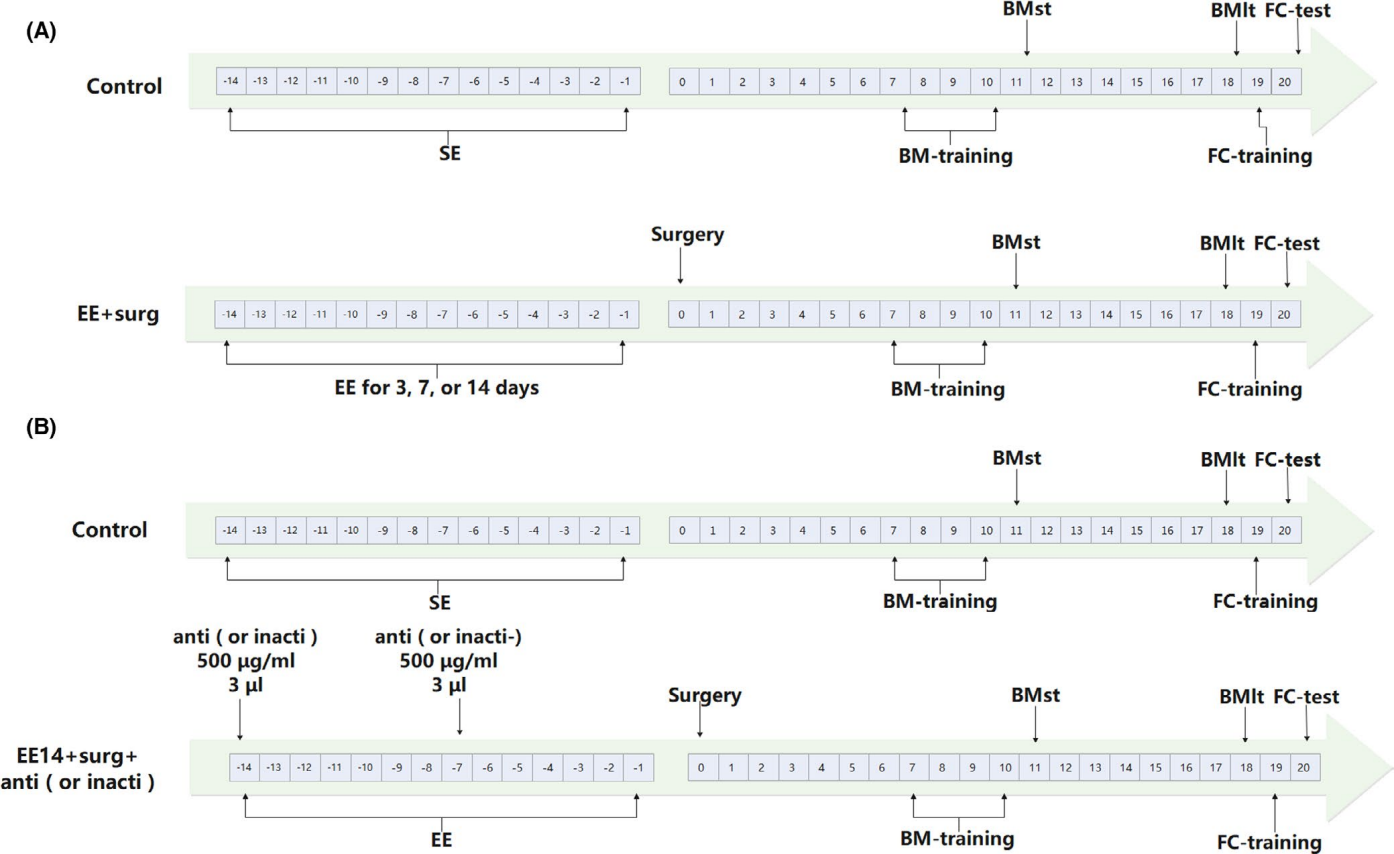
Diagram of time line of experiments. (A) Time line of experiments determining the length of EE needed for improving learning and memory in mice with surgery. (B) Time line of experiments determining the role of neuroligin 1 in the EE effects on mice with surgery. BM: Barnes maze, BMlt: Barnes maze long‐term memory, BMst: Barnes maze short‐term memory, FC: fear conditioning, inacti: heat‐inactivated anti‐neuroligin 1 antibody, anti: anti‐neuroligin 1 antibody, NLGN1: neuroligin 1

In the first experiment (Figure 1A), 8‐week‐old C57BL/6J male mice were randomly divided into 6 groups with 25 mice in each group: (1) control, (2) EE for 2 weeks, (3) standard environment (SE) before surgery, (4) EE for 3 days before surgery, (5) EE for 1 week before surgery, and (6) EE for 2 weeks before surgery. Animals were used for learning and memory tests.

In the second experiment, 8‐week‐old male C57BL/6J mice were randomly divided into 2 groups with 12 mice in each group: (1) control and (2) EE for 2 weeks. Hippocampus and cerebral cortex were harvested from these mice for biochemical tests.

In the third experiment, 8‐week‐old male C57BL/6J mice were randomly divided into 3 groups with 12 mice in each group: (1) control, (2) surgery, and (3) EE for 2 weeks before surgery. Hippocampus and cerebral cortex were harvested from these mice for biochemical tests.

In the fourth experiment (Figure 1B), 8‐week‐old C57BL/6J male mice were randomly divided into 4 groups with 14 mice in each group: (1) control, (2) EE for 2 weeks before surgery, (3) EE for 2 weeks plus receiving heat‐inactivated anti‐neuroligin 1 antibody before surgery, and (4) EE for 2 weeks plus receiving anti‐neuroligin 1 antibody before surgery. These mice were used for learning and memory tests.

Mice under the SE condition were housed three to five mice per cage in a 27.94 × 15.24 × 11.43 cm cage on a 12 h light/dark cycle with *ad libitum* access to food and water.

Mice under EE condition were housed three to five mice per cage in a large cage (43.18 × 22.86 × 19.05 cm) for 6 h (12:00–18:00) every day. The cage contained a running wheel, tunnels, shed, and various toys. These settings were changed twice per week. Mice were returned to SE for the remaining 18 h every day.

### 2.2 Anesthesia and surgery

The surgery was the right carotid artery exposure.^4^ Briefly, mice were anesthetized by 1.8 – 2% isoflurane. During the procedure, the mouse was kept on spontaneous respiration. Rectal temperature was monitored and maintained at 37°C with the aid of a heating blanket (TCAT‐2LV, Physitemp Instruments Inc., Clifton, NJ). A 1.5‐cm midline neck incision was made after incision site was infiltrated with 0.25% bupivacaine. Soft tissues over the trachea were retracted gently. One‐centimeter long right common carotid artery was carefully dissected free from adjacent tissues without any damage to vagus nerve. The wound was then irrigated and closed by using surgical suture. The surgical procedure was performed under sterile conditions and lasted around 15 min. After the surgery, all animals received a subcutaneous injection of bupivacaine. The total duration of general anesthesia was 2 h. No response to toe pinching was observed during the anesthesia.

### 2.3 Barnes maze

One week after surgery (Figure 1), mice were subjected to Barnes maze as we described before^4^, ^23^ to test their spatial learning and memory. Barnes maze is a circular platform with 20 equally spaced holes (SD Instruments). One hole was connected to a dark chamber called “target box.” Mice were placed in the middle of the platform and encouraged to find the target box by aversive noise (85 dB) and bright light (200 W) shed on the platform. They had a spatial acquisition phase that included training for 4 days with four trials per day, 3 min per trial, and 2 h between each trial. The time to find the target box within 3 min was recorded as latency. If the animal could not find the target box within 3 min, the latency for that trial was recorded as 3 min. The animal was allowed to stay in the target box for 1 min. The reference memory of the mice was tested on day 5 and day 12, respectively. One trial on each of these 2 days was performed. The mice were not subjected to any tests during the period from day 5 to day 12. The latency to find the target box during each trial was recorded with the assistance of ANY‐Maze video tracking system (SD Instruments).

### 2.4 Fear conditioning test

One day after the Barnes maze test, mice were subjected to the fear conditioning test using the Freeze Monitor from San Diego Instruments (San Diego, CA) in the same way as we described before.^4^, ^23^ Briefly, each animal was placed in a test chamber wiped with 70% alcohol and subjected to three tone‐foot shock pairings (tone: 2 kHz, 85 db, 30 s; foot shock: 0.7 mA, 2 s) with an intertrial interval of 1 min in a relatively dark room. The animal was removed from this test chamber 30 s after the conditioning training. The animal was placed back in the chamber 24 h later for 6 min in the absence of tone and shock. The amount of time with freezing behavior was recorded in this 6 min. The animal was placed 2 h later in a test chamber that had a different context and smell environment from the first test chamber (this second chamber was wiped with 1% acetic acid) in a relatively light room. After a 3‐min acclimatization time, the auditory stimulus was turned on for three cycles, each cycle for 30 s followed by a 1‐min inter‐cycle interval (4.5 min in total). The freezing behavior in the 4.5‐min period was recorded. Freezing behavior was defined as absence of all movements except for respiration. Freezing behavior as seen in the video was scored by an observer who was blind to group assignment. These tests determine hippocampus‐dependent (context‐related) and hippocampus‐independent (tone‐related) learning and memory.^24^

### 2.5 Neuroligin 1 antibody injection

In the fourth experiment, some mice received intracerebroventricular injection of 3 μl (500 μg/ml) anti‐neuroligin 1 antibody (catalog number: 129111, Synaptic Systems, Germany). Some others received the injection of 3‐μl heat‐inactivated (5 min at 100°C) anti‐neuroligin 1 antibody. Each mouse received two injections: one was 2 weeks before surgery and the other one was 1 week before surgery. This anti‐neuroligin 1 antibody reacts with rat and mouse neuroligin 1 and does not have cross‐reactivity to neuroligins 2 and 3 as tested in a previous study.^25^

The intracerebroventricular injection was performed with the aid of a stereotactic apparatus (SAS‐5100, ASI Instruments, Warren, MI) using the following coordinates: 1.0 mm posterior to bregma, 1.5 mm lateral from midline, and 4.0 mm ventral from the surface of the skull. After the injection, the needle was kept in place for 1 min to prevent backflow of the injected solution. Mice were anesthetized by 1.8 – 2% isoflurane. The anti‐neuroligin 1 antibody was injected intracerebroventricularly instead of being injected directly into the hippocampus because of the consideration that intracerebroventricular injection would avoid direct injection injury to hippocampus and injection to one side of ventricle would be enough for the antibody to affect bilateral hippocampus and other brain tissues.

### 2.6 Tissue harvest

For western blotting and assay of HDAC activity, brain tissues were harvested one day after the completion of the 2 weeks of EE or one day after surgery. For this purpose, mice were anesthetized with isoflurane. After trans‐cardiac perfusion with ice‐cold normal saline, hippocampus and cerebral cortex were isolated immediately on ice and then stored at −80^0^C until used.

### 2.7 HDAC activity assay

HDAC activity in the hippocampus and cerebral cortex was detected with HDAC assay kit (Colorimetric Assay Kit, catalog number: GTX85529, Genetex, CA) according to the manufacturer's instruction. Briefly, brain tissues were homogenized, and 50‐μg sample was added to 85 μl water in each well. Background reading was performed on water. Positive control was 10 μl HeLa nuclear extract with 75 μl water. Negative control was 50 μg sample with 83 μl water and 2 μl trichostatin A. The OD values were read in a microplate reader (Bio RAD 680, Japan) at 415 nm.

### 2.8 Western blotting

Western blotting was performed as previously described.^26^ In brief, protein concentrations of samples prepared by homogenizing brain tissues were determined using the BCA protein assay (Bio‐Rad, Hemel Hempstead, Herts, UK). Twenty microgram proteins of each sample were subjected to western blotting analysis using the following primary antibodies: rabbit polyclonal recombinant anti‐histone H3 (acetyl k14) antibody (EP964Y) (catalog number: ab52946, Abcam, Cambridge, MA) at 1:1000 dilution; rabbit polyclonal recombinant anti‐histone H4 (acetyl k5) antibody (EP1000Y) (catalog number: ab51997, Abcam) at 1:1000 dilution; rabbit polyclonal anti‐neuroligin 1 antibody (catalog number: 129111, Synaptic Systems, Germany) at 1:1000 dilution, and rabbit polyclonal anti‐α‐Tubulin antibody (cell signaling Technology Inc.) at 1:1000 dilution. Images were scanned by an Image Master II scanner (GE Healthcare, Milwaukee, WI) and analyzed using ImageQuant TL software v2003.03 (GE Healthcare). The band signals of the interesting proteins were normalized to those of the corresponding α‐tubulin and expressed as fractions of control sample on the same gels.

### 2.9 Statistical analysis

All data were analyzed by SigmaStat (Systat Software, Inc.). The Kolmogorov‐Smirnov test was used to test the normality of the data. Data in normal distribution are present as mean ±SD with the presence of individual animal data in the bar graphs. Data in non‐normal distribution are presented as median ± interquartile range with the presence of individual animal data in the bar graphs. The results were analyzed by using Student's *t*‐test, one‐way analysis of variance, or one‐way analysis of variance on ranks followed by the Tukey test as appropriate. Two‐way or one‐way repeated measures analysis of variance was used to compare data of Barnes maze training sessions between groups and within one group, respectively. Differences were considered significant at a *p* < 0.05.

## 3 RESULTS

### 3.1 Preoperative EE improved learning and memory in surgery mice

**FIGURE 2:**
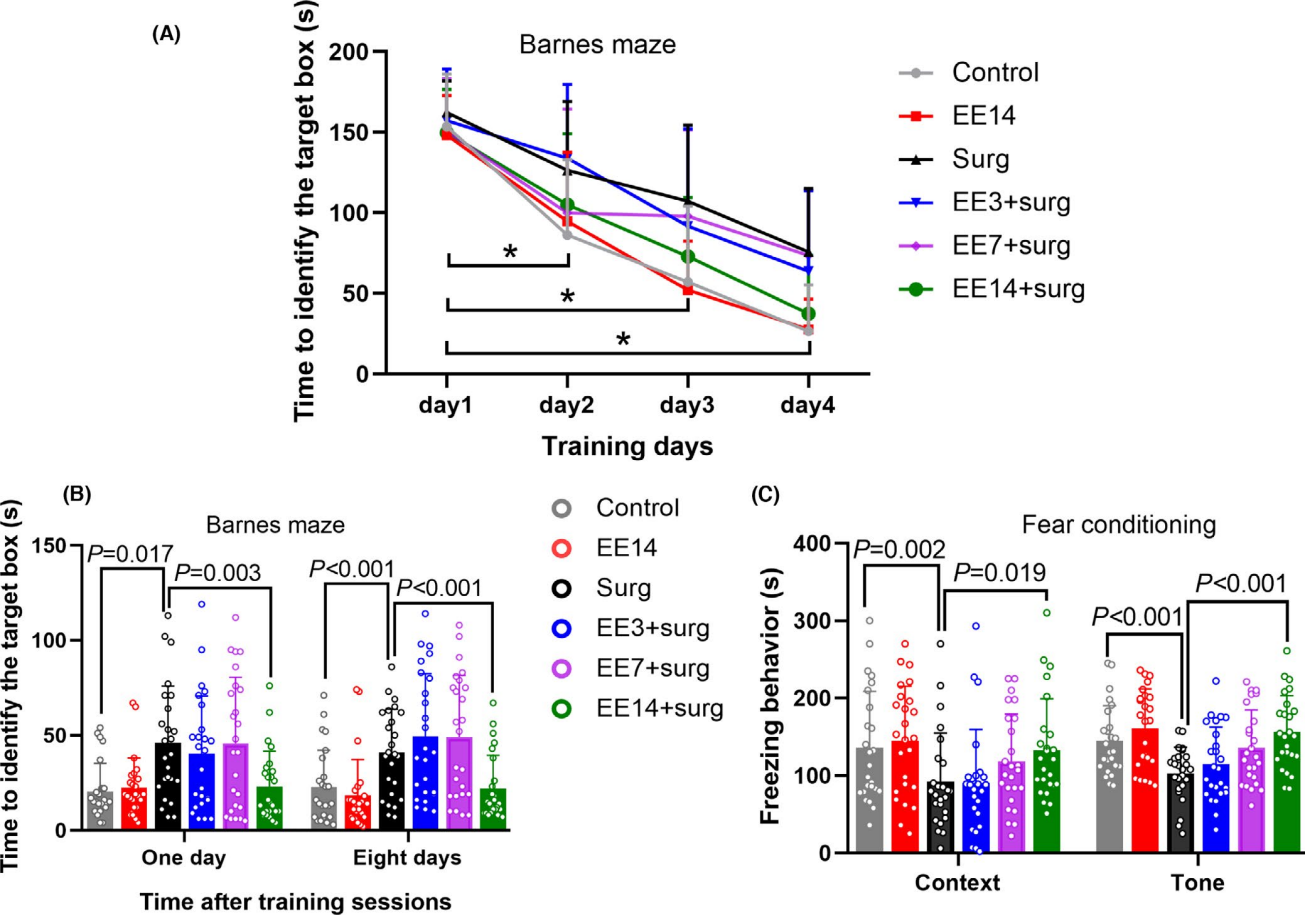
EE *reduced learning and memory impairment in mice with surgery*. Mice were subjected to various experimental conditions and tested in the Barnes maze and fear conditioning paradigms starting 7 days after surgery. (A) Performance in the training sessions of Barnes maze test. (B) Performance in the memory phase of Barnes maze test. (C) Performance in fear conditioning paradigm. Results are median ± interquartile range (n = 25) with the presence of individual animal data in the bar graphs. Results were analyzed by two‐way or one‐way repeated measures analysis of variance for panel A and by one‐way analysis of variance on ranks followed by the Tukey test for panels B and C. * indicates *p* < 0.05 for the comparisons of values of day 1 with those of day 2, values of day 1 with those of day 3 and values of day 1 with those of day 4 for all groups except for the comparisons of values of day 1 with those of day 2 for the EE14 and Surg+EE3 groups. EE3: EE for 3 days, EE7: EE for 7 days, EE14: EE for 14 days, Surg: surgery

The time needed for mice in all six groups to identify the target box was decreased with increased training sessions (Figure 2A). Surgery was a significant factor to affect the time needed to identify the target box in the Barnes maze test [F(1,48) =21.302, *p* < 0.001, surgery group vs. control group]. Although EE did not affect the performance of control mice [F(1,48) =0.00027, *p* = 0.987], EE was a significant factor to affect the performance of mice with surgery in the training sessions when EE at three levels (for 3, 7, and 14 days, respectively) was considered [F(3,96) =2.721, *p* = 0.049]. There was an interaction between training sessions/days and EE lengths [F(9,288) =2.705, *p* = 0.005] with difference in time needed to identify the target box between surgery mice and surgery mice plus 2‐week EE on days 2, 3, and 4 of the training sessions. Consistent with this, EE for 2 weeks, but not EE for 3 days or 1 week, was a significant factor to affect the performance of mice in the training sessions [F(1,48) =11.694, *p* = 0.001]. Similarly, surgery increased the time for mice to identify the target box at one or eight days after the training sessions. EE for 2 weeks, but not EE for 3 days or 1 week, reduced this time for surgery mice (Figure 2B). Surgery also reduced the freezing behavior in both context‐ and tone‐related fear conditioning tests. EE for 2 weeks attenuated this reduction (Figure 2C). These results suggest that surgery impairs learning and memory and that EE for 2 weeks, but not EE for 3 days and 7 days, attenuated surgery‐induced impairment of learning and memory. Since the performance of context‐related fear conditioning is hippocampus‐dependent and tone‐related fear conditioning may not be hippocampus‐dependent,^24^ our results suggest that hippocampus‐dependent and hippocampus‐independent learning and memory may be impaired by surgery and that this impairment is attenuated by EE.

### 3.2 EE attenuated surgery‐induced reduction of neuroligin 1 possibly via attenuating epigenetic dysregulation

**FIGURE 3:**
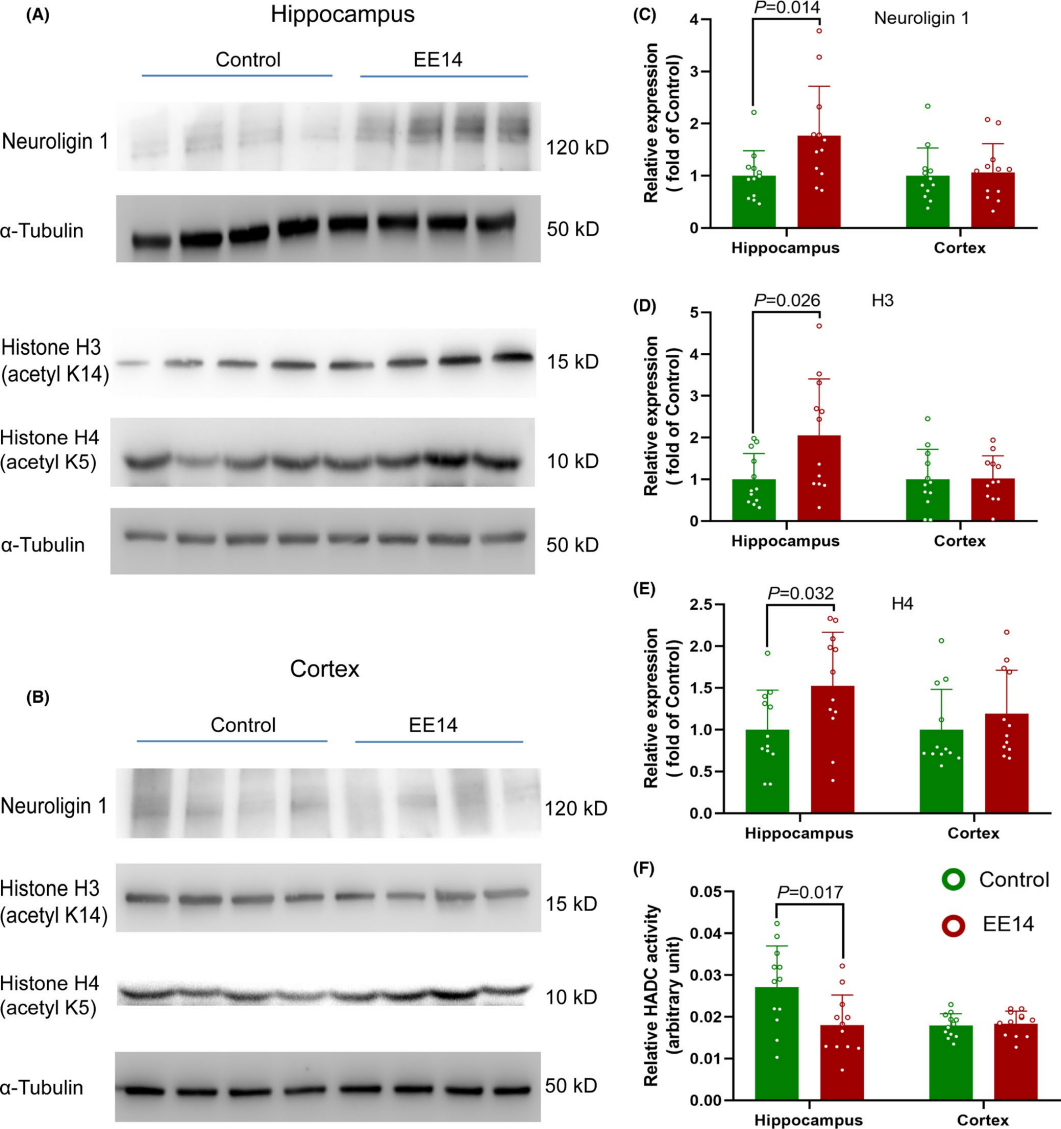
EE *increased neuroligin 1 in the hippocampus*. Mice had EE or SE for 14 days. Their hippocampus and cerebral cortex were harvested 24 h after the completion of EE. (A) Representative images of western blotting of hippocampal samples. (B) Representative images of western blotting of cerebral cortical samples. (C) Quantitative data of neuroligin 1. (D) Quantitative data of acetylated histone H3. (E) Quantitative data of acetylated histone H4. (F) HDAC activity. Results are mean ± S.D. (*n* = 12) with the presence of individual animal data in the bar graphs. Results were analyzed by t‐test. EE14: EE for 14 days

**FIGURE 4:**
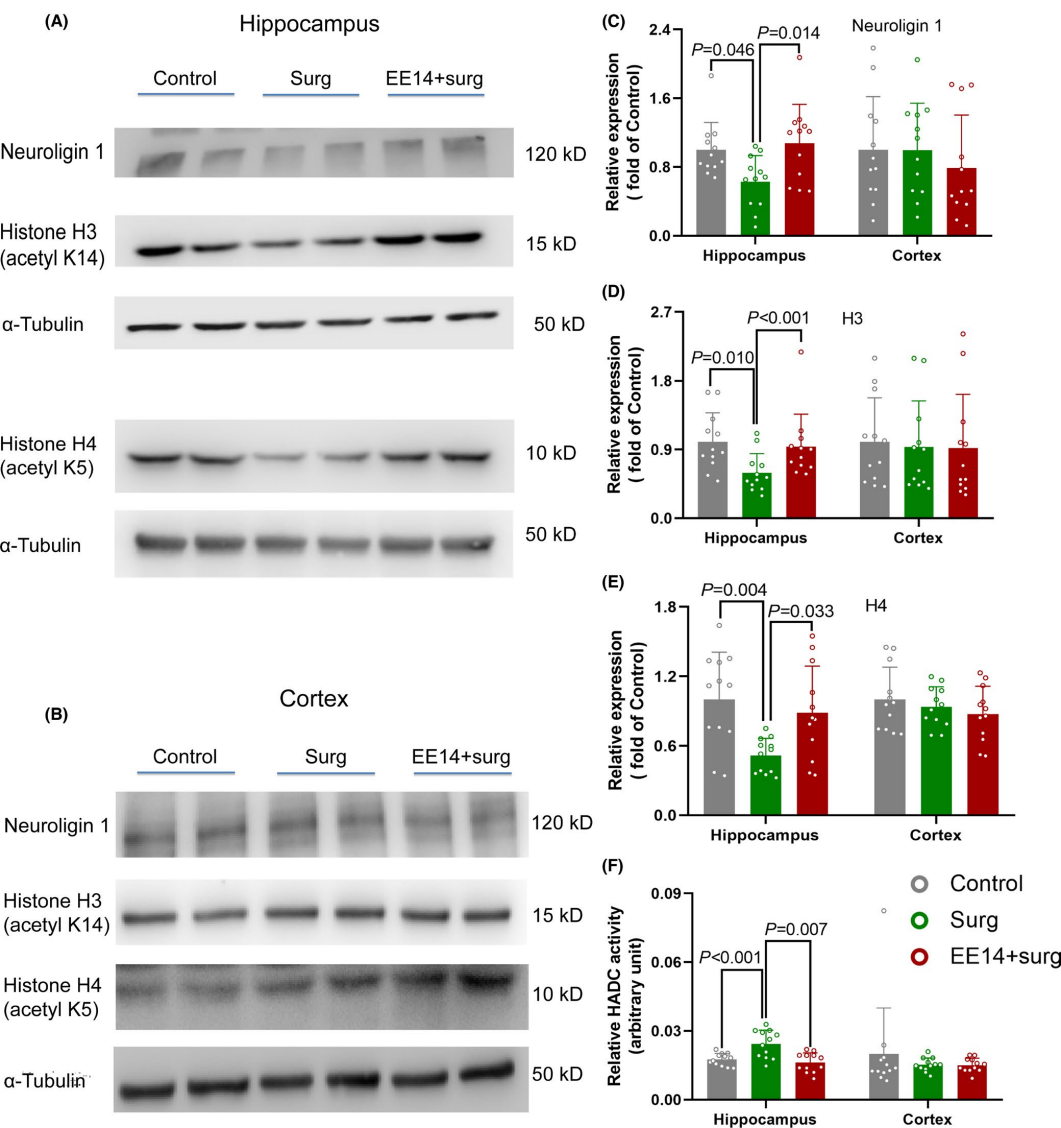
EE *attenuated the decrease of neuroligin 1 in the hippocampus of mice with surgery*. Mice had EE or SE for 14 days before they had surgery. Their hippocampus and cerebral cortex were harvested 24 h after the surgery. (A) Representative images of Western blotting of hippocampal samples. (B) Representative images of western blotting of cerebral cortical samples. (C) Quantitative data of neuroligin 1. (D) Quantitative data of acetylated histone H3. (E) Quantitative data of acetylated histone H4. (F) HDAC activity. Results are mean ± S.D. (*n* = 12) with the presence of individual animal data in the bar graphs. Results were analyzed by one‐way analysis of variance followed by the Tukey test. EE14: EE for 14 days

EE increased neuroligin 1, acetylated H3, and acetylated H4 histone in the hippocampus but not in the cerebral cortex of control mice. Consistent with these results, HDAC activity in the hippocampus was decreased by EE (Figure 3). Surgery decreased neuroligin 1, acetylated H3, and acetylated H4 histone in the hippocampus but not in the cerebral cortex. Consistent with our previous study,^7^ surgery increased HDAC activity in the hippocampus. EE preserved the expression of neuroligin 1, acetylated H3, and acetylated H4 histone in the hippocampus and reduced HDAC activity in the hippocampus of surgery mice (Figure 4). These results suggest that surgery reduces neuroligin 1 and induces epigenetic dysregulation and that EE attenuates these surgical changes.

### 3.3 Neutralizing neuroligin 1 blocked the normalization of learning and memory in surgery mice with EE

**FIGURE 5:**
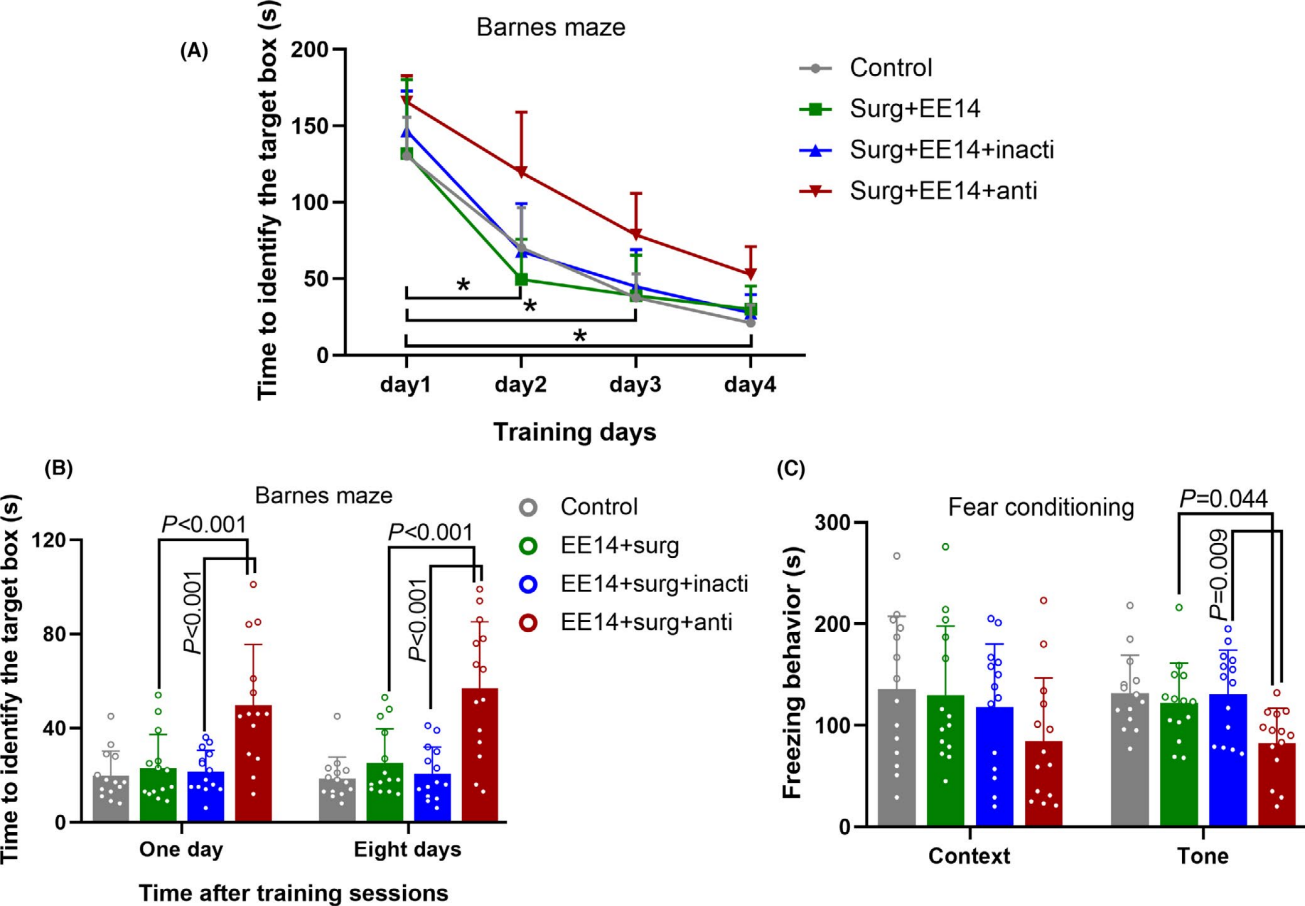
Neuroligin *1 contributed to the protection of EE against surgery*‐*induced learning and memory impairment*. Mice were subjected to various experimental conditions and tested in the Barnes maze and fear conditioning paradigms starting 7 days after surgery. (A) Performance in the training sessions of Barnes maze test. (B) Performance in the memory phase of Barnes maze test. (C) Performance in fear conditioning paradigm. Results are median ±interquartile range (n = 14) with the presence of individual animal data in the bar graphs. Results were analyzed by two‐way or one‐way repeated measures analysis of variance for panel A and by one‐way analysis of variance on ranks followed by the Tukey test for panels B and C. * indicates *p* < 0.05 for the comparisons of values of day 1 with those of day 2, values of day 1 with those of day 3 and values of day 1 with those of day 4 for all groups except for the comparisons of values of day 1 with those of day 2 for the control and EE+surg+anti groups. EE14: EE for 14 days, Surg: surgery, Inacti: heat‐inactivated anti‐neuroligin 1 antibody, Anti: anti‐neuroligin 1 antibody

To determine the role of preserved neuroligin 1 expression in the protection of EE against surgical effects, mice with surgery and EE received an anti‐neuroligin 1 antibody. This antibody, but not the heat‐inactivated antibody, was a significant factor to affect the time needed for surgery mice with EE to identify the target box during training sessions in the Barnes maze test [F(1,26) =28.302, *p* < 0.001 for comparison between mice with surgery plus EE and mice with surgery plus EE and antibody; F(1,26) =1.837, *p* = 0.187 for comparison between mice with surgery plus EE and mice with surgery plus EE and inactivated antibody]. The comparison between mice with surgery plus EE plus antibody and mice with surgery plus EE plus inactivated antibody was different [F(1,26) =24.712, *p* < 0.001] (Figure 5A). The antibody increased the time for mice with surgery plus EE to identify the target box one or eight days after the training sessions in the Barnes maze test (Figure 5B) and reduced context‐ and tone‐related freezing behavior of these mice in the fear conditioning test (Figure 5C). These results suggest that neuroligin 1 plays a role in the EE effects on learning and memory of surgical mice.

## 4 DISCUSSION

Consistent with our previous studies,^27^, ^28^ surgery induced learning and memory impairment in the mice as indicated by their poor performance in the Barnes maze and fear conditioning tests. A previous study has shown that EE for 2 weeks before surgery‐reduced learning and memory impairment in rats.^13^ However, the length of EE needed for this effect is not known. Our study showed that the length of preoperative EE needed to be effective in reducing POCD is 2 weeks because EE for 3 or 7 days did not improve the learning and memory in mice with surgery. These results suggest that preoperative EE needs to be a certain length to provide the protective effects.

To determine molecular mechanisms for EE to reduce POCD, we focused on neuroligin 1. There are three neuroligins in rodents.^29^ Neuroligin 1 is expressed in excitatory synapses, neuroligin 2 is in inhibitory synapses and neuroligin 3 is found in both types of synapses.^15^, ^16^, ^29^ Those neuroligins are in the postsynaptic membrane and interact with β‐neurexins in the presynaptic membrane to pull the presynaptic and postsynaptic membrane close enough to form synapses. Neuroligins also recruit and stabilize the protein complex to postsynaptic membrane. Thus, these proteins are critical for synapse formation.^15^, ^16^, ^29^ Our previous studies have shown that surgery reduces dendritic arborization and spine density.^7^, ^30^ Therefore, it is possible that neuroligins are molecular targets for the surgery effects. We focused on neuroligin 1 because it has been shown to be involved in long‐term potentiation formation,^17^ and neuroligin 1 knockdown decreases dendritic spine density and excitatory synaptic currents in the hippocampus.^31^ Our results showed that surgery reduced neuroligin 1 in the hippocampus. EE attenuated this reduction and the impairment of learning and memory in mice with surgery. An anti‐neuroligin 1 antibody given intracerebroventricularly impaired learning and memory of mice with surgery plus EE but the same antibody that was heat‐inactivated did not have these effects. These results suggest that neuroligin 1 plays an important role in POCD and the effects of EE in reducing POCD. Interestingly, neuroligin 1 silencing leads to fearful memory storage deficit.^17^ Consistent with this previous finding, surgery mice had a deceased level of neuroligin 1and poor performance in fear conditioning test in our study. Also, neuroligin 1 genotype variations are associated with different levels of anxiety and fear in patients,^32^ suggesting a role of neuroligin 1 in emotional regulation. Although there is no evidence to suggest that patients with POCD have an altered level of anxiety or depression,^3^ careful investigation on potential changes in emotion after surgery in pre‐clinical and clinical studies may identify additional feature of POCD.

We have shown that surgery activates HDAC to reduce growth factor expression, which then leads to impaired dendritic arborization and spine density.^7^ Similarly, this current study showed that surgery increased HDAC activity and reduced acetylated H3 and H4 histone. These effects were attenuated by EE. Reduced acetylation in histone has been generally considered to inhibit the expression of genes because acetylated histone will allow easy access of transcription factors to the DNA to initiate transcription of genes.^33^, ^34^ Thus, a possible mechanism for surgery to regulate neuroligin 1 is that surgery activates HDAC, which then reduces the expression of neuroligin 1. EE can attenuate this epigenetic dysregulation caused by surgery to preserve neuroligin 1 expression. Consistent with this possibility, a previous study has shown that amyloid fibril induces neuroinflammation that then activates HDAC to reduce neuroligin 1 to impair learning and memory in rats.^22^ Since neuroinflammation has been considered a major mechanism for POCD and neuroinflammation impairs learning and memory,^4^, ^9^, ^35^, ^36^ a possible pathway for POCD is surgery‐neuroinflammation‐HDAC activation‐neuroligin 1 decrease‐synapse impairment‐learning and memory impairment. EE can disrupt this pathway to attenuate POCD.

Interestingly, the effects of surgery and EE on neuroligin 1 expression, HDAC activity, and acetylated H3 and H4 histone expression in the hippocampus, a brain region that is involved in learning and memory,^24^ were obvious. However, these effects appear to be minimal in the cerebral cortex. The reasons for this difference are not clear, especially in the context that surgery causes neuroinflammation in cerebral cortex.^4^, ^9^, ^35^, ^37^ It may be possible that there are mechanisms that antagonize the effects of inflammation in the cerebral cortex on the epigenetic regulation of gene expression. Another possibility is that inflammation does not alter the enzymatic activity for epigenetic regulation in the cortex. Consistent with this possibility, surgery did not change the HDAC activity in these tissues. Regardless, our results suggest a brain region difference in the effects of surgery and EE on neuroligin 1 expression and epigenetic regulation of gene expression.

Our current study focused on the role of neuroligin 1 in POCD and in the effects of EE on POCD development. Our previous study has shown that surgery increases HDAC activity and reduced brain‐derived neurotrophic factor in the hippocampus.^7^ Our most recent study has shown that surgery decreases postsynaptic density protein 95 and synapsin 1, two synaptic proteins, and preoperative exercise attenuates this decrease.^38^ Thus, surgery can reduce multiple proteins in addition to neuroligin 1. These affected proteins include other synaptic proteins, such as synapsin 1 and postsynaptic density 95.

We used common carotid artery exposure as the surgical model. Many other surgical models, such as laparotomy and orthopedic surgery,^7^, ^8^ have been used for POCD research. Consistent with this current study, our previous study has shown that mice with laparotomy have increased HDAC activity and reduced brain‐derived neurotrophic factor. In addition, inhibiting HDAC preserves brain‐derived neurotrophic factor expression and reduces POCD.^7^ Thus, similar mechanisms may be in play for the regulation of gene expression and the development of POCD in different surgical models.

Our previous and current studies and one study from another group have shown that EE practiced before and after surgery effectively reduced learning and memory impairment after surgery in rodents.^12^, ^13^, ^14^ EE is a non‐pharmacological approach and will avoid side effects of medications. This feature is advantageous, especially in elderly patients who are often on multiple medications. Thus, EE may be a useful approach to reduce POCD if its effectiveness is shown in humans.

Our study has limitations. The study was performed in male mice. Future studies shall determine whether EE can reduce POCD in female mice. However, EE applied to middle‐aged mice reduces aging‐induced learning and memory impairment, which is not sex‐dependent.^39^ Also, unlike in our previous study,^7^ we did not use a HDAC inhibitor to determine whether the activation of HDAC was a cause for the reduced neuroligin 1 in mice with surgery. However, EE reduced the activation of HDAC and preserved neuroligin 1 expression in mice with surgery. In addition, a previous study has shown the regulation of neuroligin 1 expression by HDAC.^22^ Thus, the increased activity of HDAC may be a course of the decreased neuroligin 1 expression.

## 5 CONCLUSION

Our results suggest that preoperative EE for 2 weeks is needed for reducing POCD in mice. This effect may be mediated by inhibiting the activation of HDAC and preserving neuroligin 1 expression in mice with surgery.

## CONFLICT OF INTERESTS

The authors declare no competing interests.

## AUTHOR CONTRIBUTIONS

ZZ conceived the project. JM and ZZ designed the study, JM, ZL, and HW performed the experiments. JM did the initial data analysis and drafted the Methods and Materials Section. ZZ performed the final data analysis and wrote the manuscript.

## CONSENT FOR PUBLICATION

Not applicable.

## Supporting information

Supplementary Material

## Data Availability

Available from the corresponding author upon request.
