# Supplementary material for: Preoperative environment enrichment preserved neuroligin 1 expression possibly via epigenetic regulation to reduce postoperative cognitive dysfunction in mice
Source: CNS Neurosci Ther. 2021 Dec 9;28(4):619–29. doi: 10.1111/cns.13777 (PMC8928916; doi:10.1111/cns.13777)

Fig3-A
Neuroligin 1 on Hippocampus

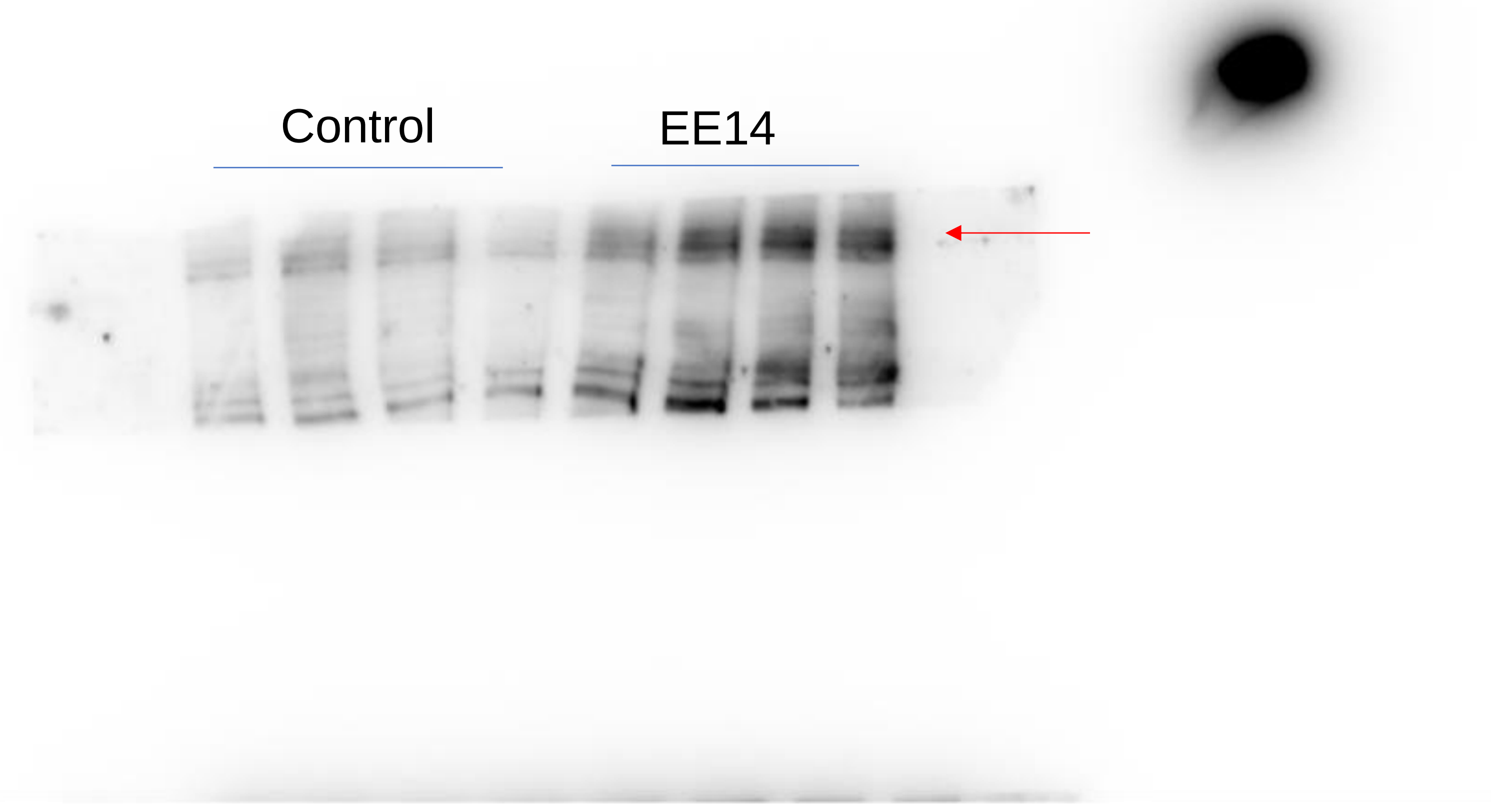

Fig3-A
α-Tubulin on Hippocampus

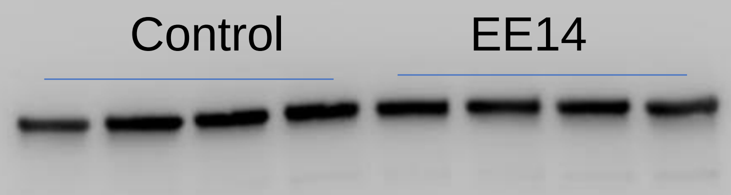

Fig3-A
Histone H3 on Hippocampus

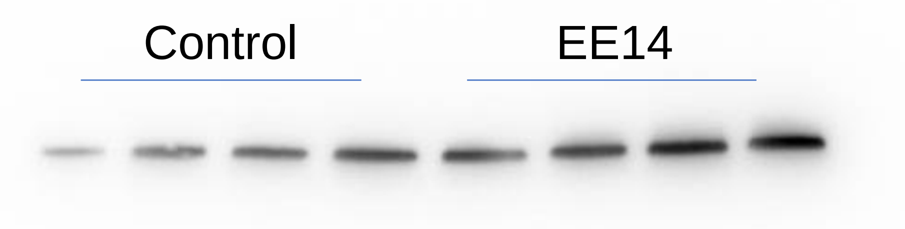

Fig3-A
Histone H4 on Hippocampus

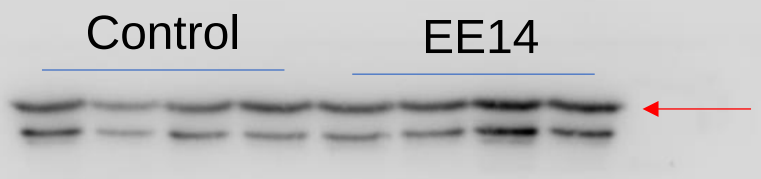

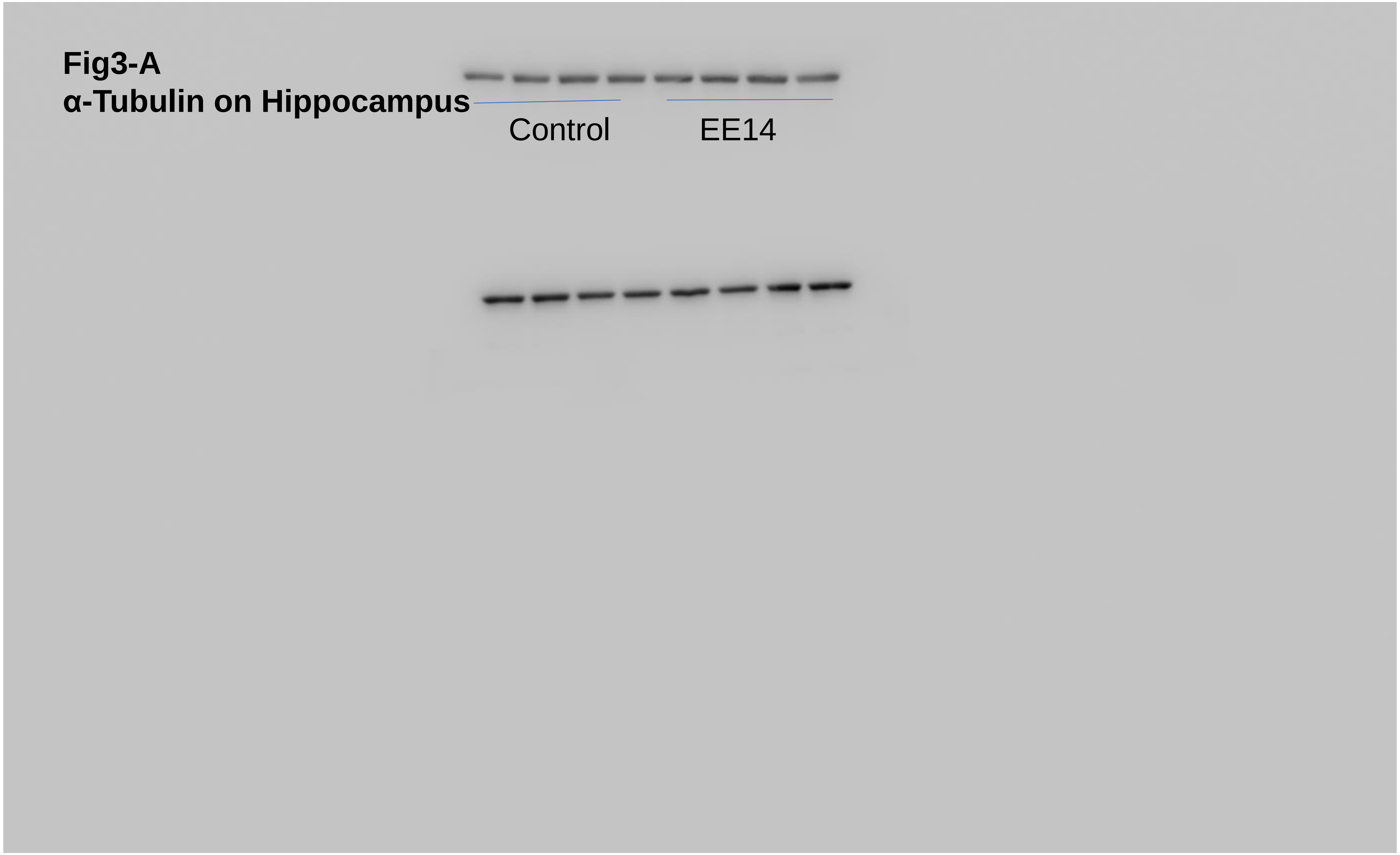
Fig3-A
α-Tubulin on Hippocampus
Control
EE14

Fig3-B
Neuroligin 1 on Cortex

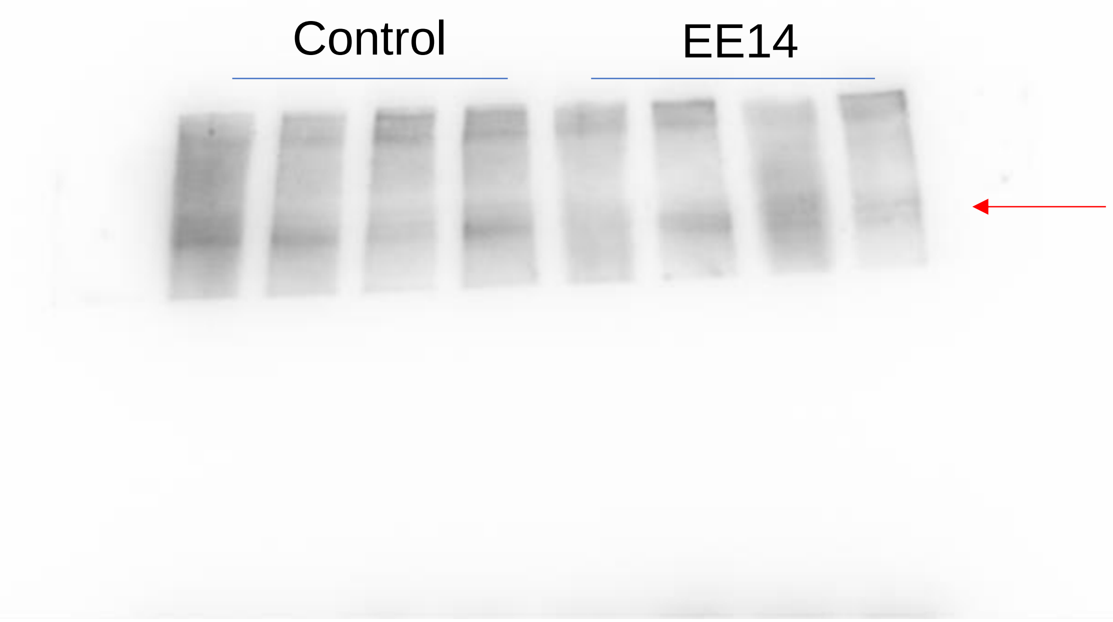

Fig3-B
Histone H3 on Cortex

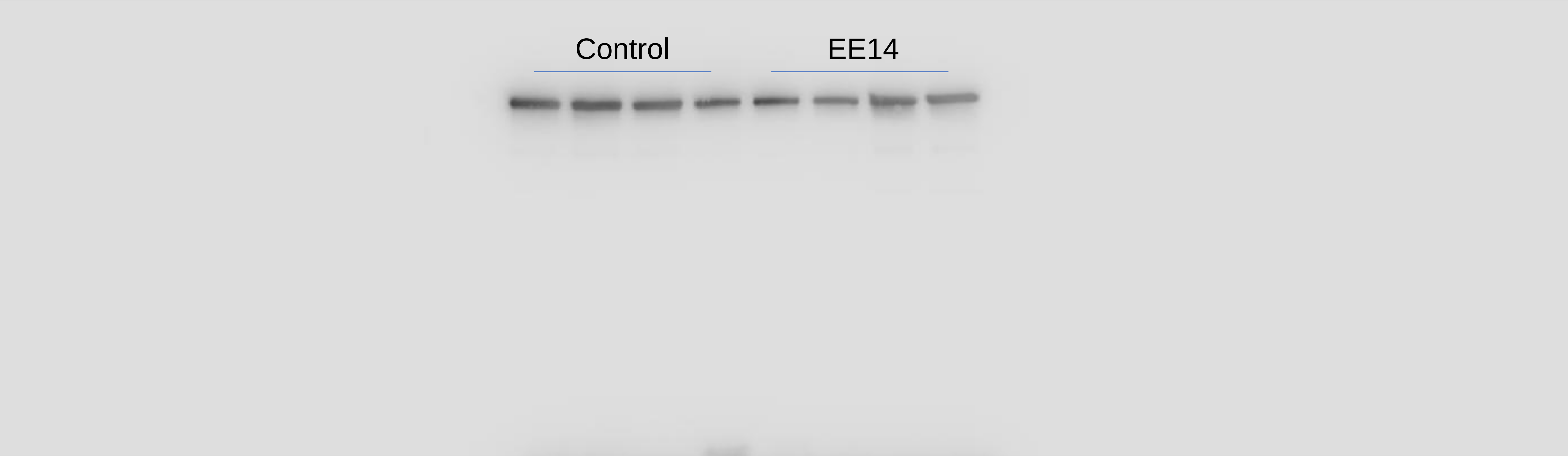

Fig3-B
Histone H4 on Cortex

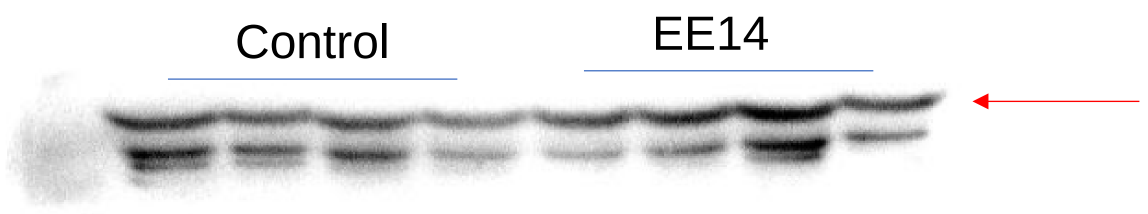

Fig3-B
α-Tubulin on Cortex

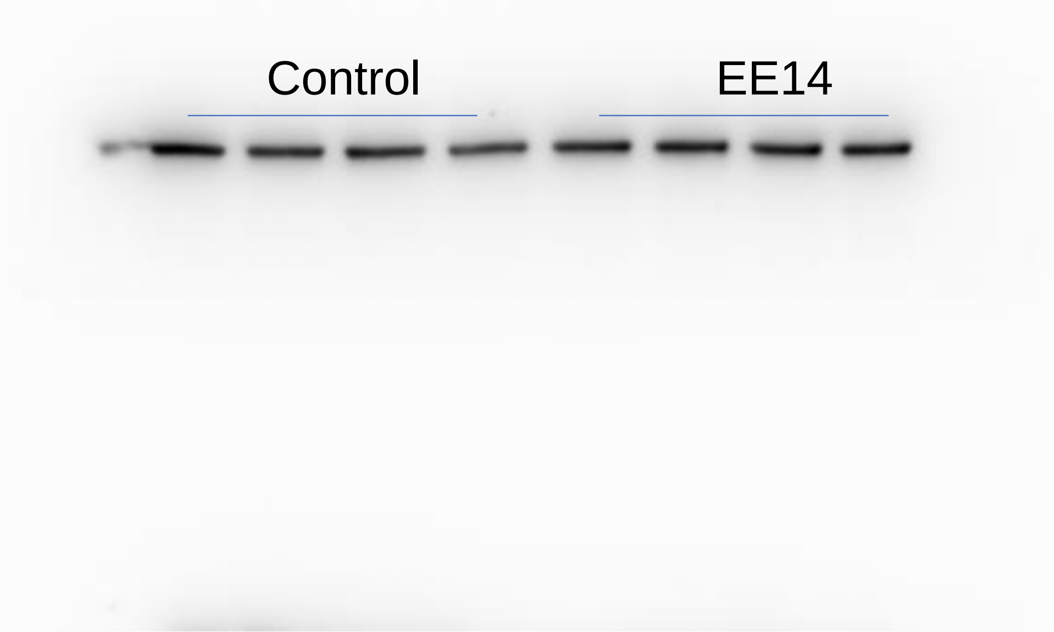

Fig4-A

Neuroligin 1 on Hippocampus

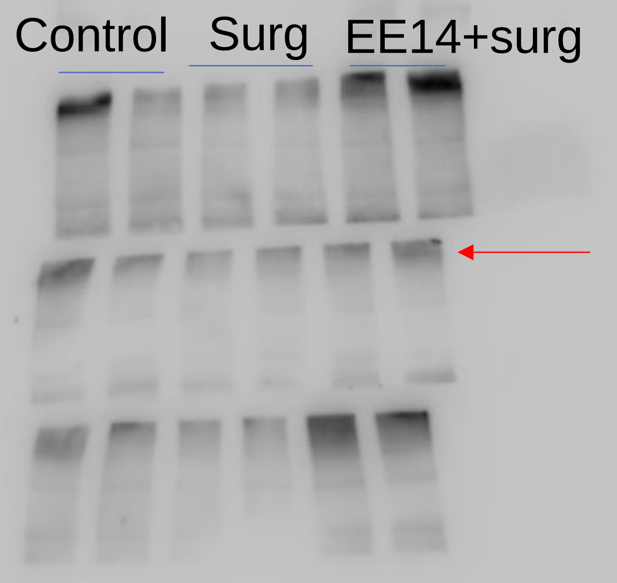

Fig4-A
Histone H3 on Hippocampus

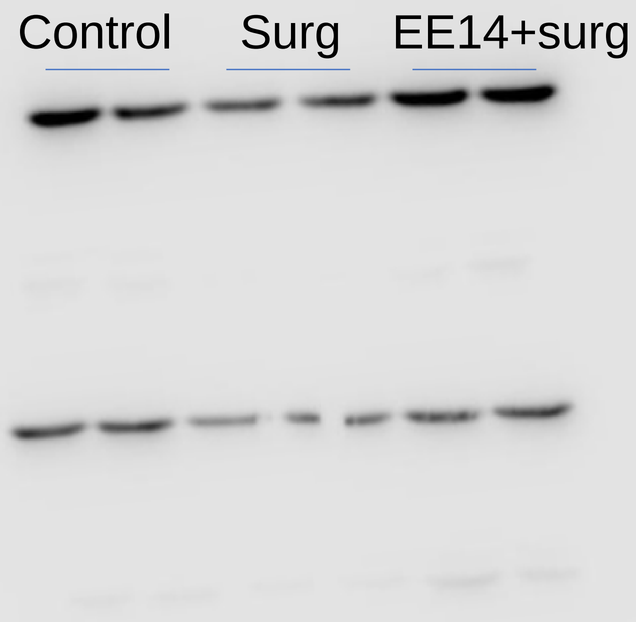

Fig4-A
α-Tubulin on Hippocampus

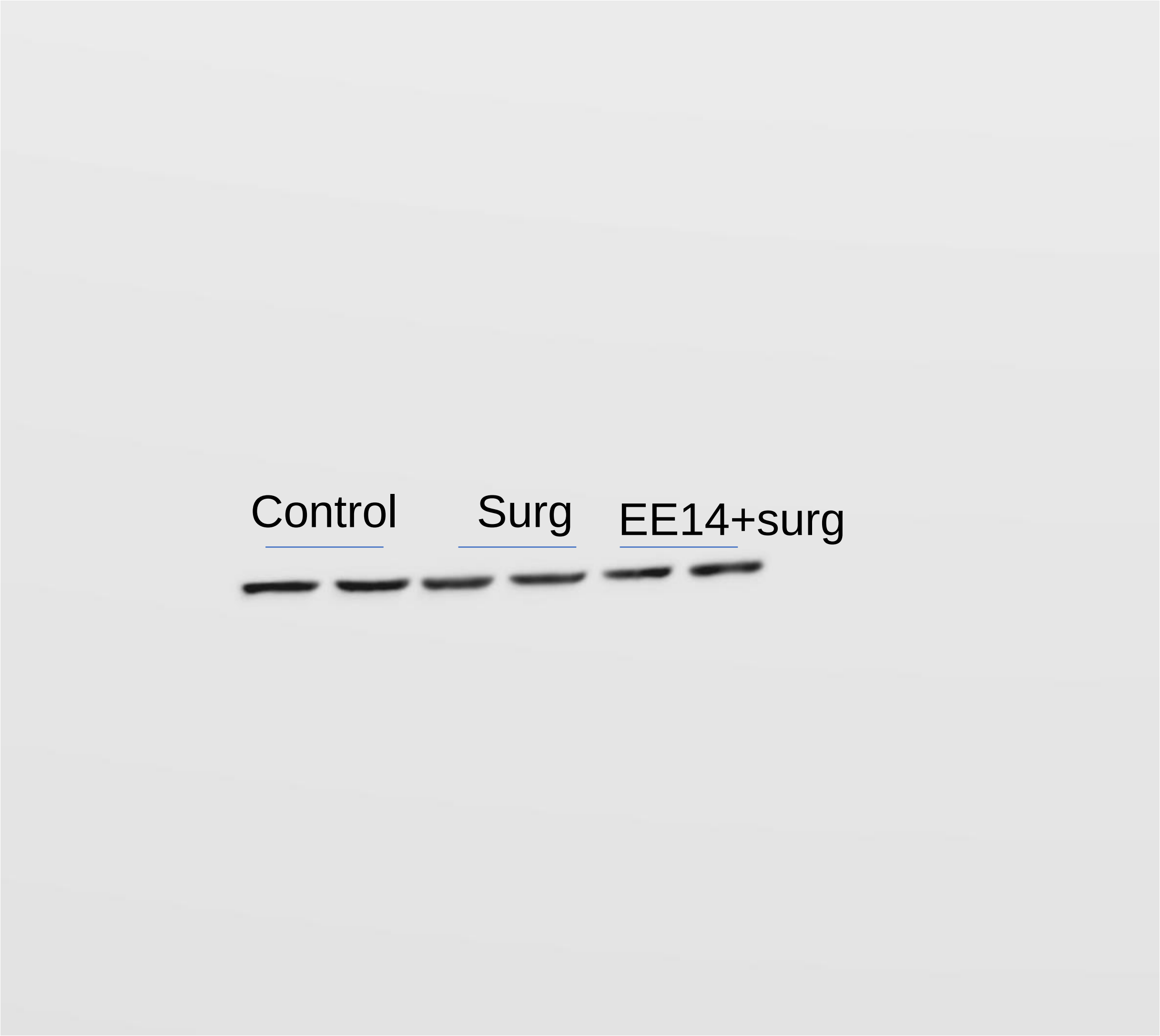

Fig4-A
Histone H4 on Hippocampus

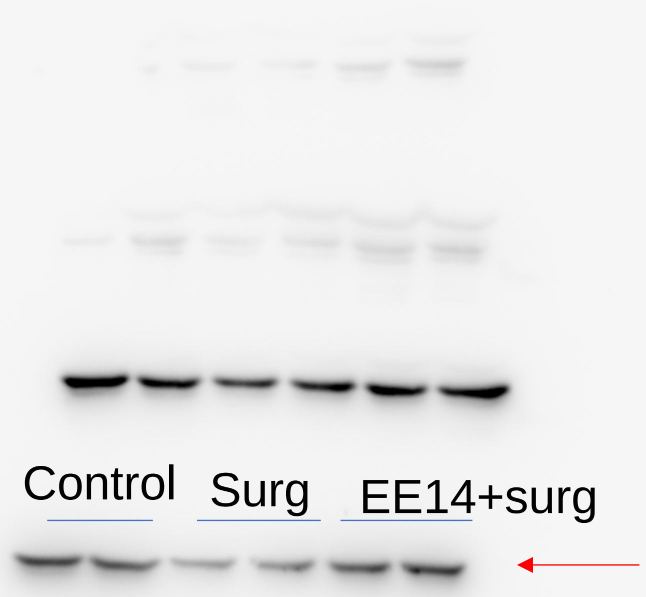

Fig4-A
α-Tubulin on Hippocampus

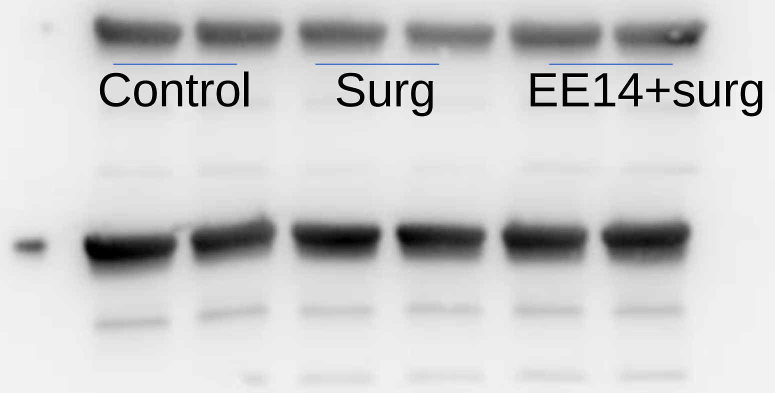

Fig4-B
Neuroligin 1 on Cortex

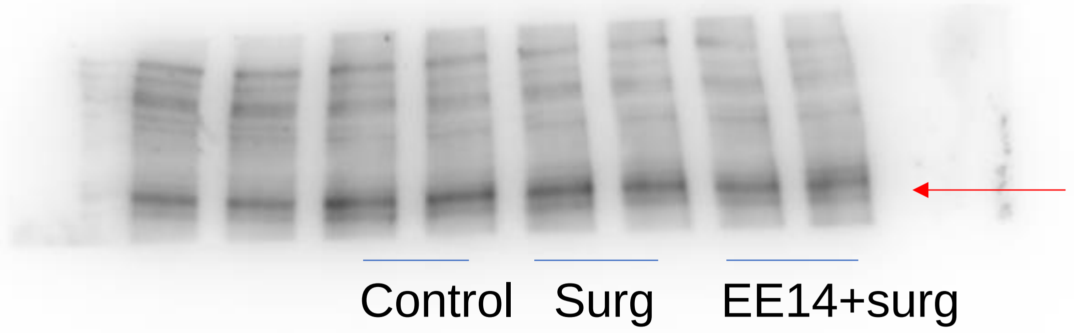

Fig4-B
Histone H3 on Cortex

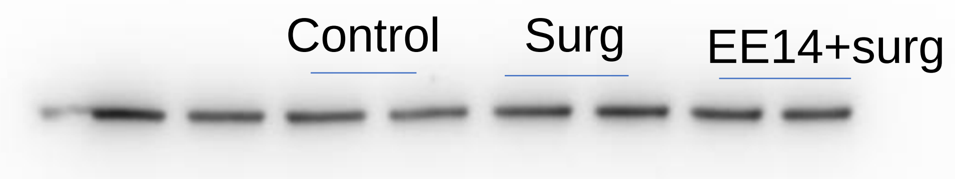

Fig4-B
Histone H4 on on Cortex

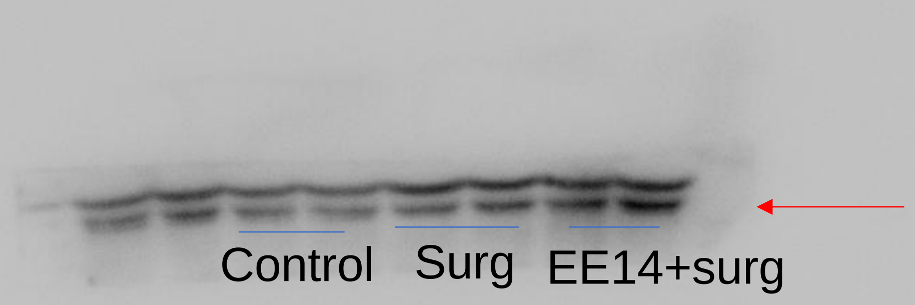

Fig4-B
α-Tubulin on Cortex

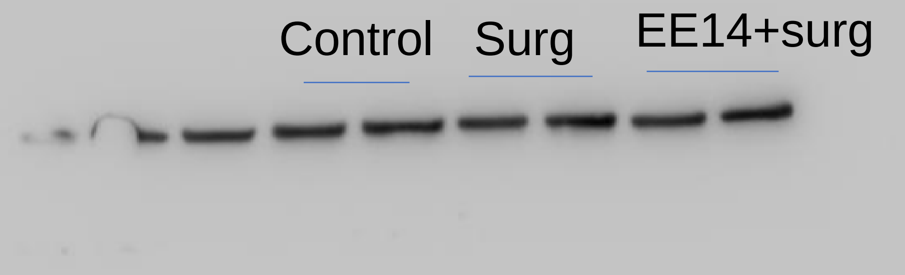

Supplement: Supplementary file 1 — Supplementary Material [file CNS-28-619-s001.pdf]
